# Diagnostic performance of generative pretrained transformer -4 with vision technology versus board-certified dermatologists: A comparative analysis using dermoscopic and clinical images

**DOI:** 10.1016/j.jdin.2024.10.006

**Published:** 2024-11-25

**Authors:** Brandon R. Block, Camille M. Powers, Annie Chang, Caroline Campbell, Austin J. Piontkowski, Jeremy Orloff, Melissa A. Levoska, Ahuva Cices, Justine Fenner, Jordan Talia, Jonas A. Adalsteinsson, Jonathan Ungar, Nicholas Gulati

**Affiliations:** Department of Dermatology, Icahn School of Medicine at Mount Sinai, New York, New York

**Keywords:** artificial intelligence, basal cell carcinoma, ChatGPT, clinical research, dermoscopy, general dermatology, generative pretrained transformer, image analysis, large language model, large multimodal model, machine learning, medical dermatology, melanoma, skin cancer, squamous cell carcinoma

*To the Editor:* The incorporation of multimodal features into OpenAI’s generative pretrained transformer (GPT) has revolutionized AI. Text- and vision-based data, formerly confined to distinct domains of machine learning, can now be paired to generate unprecedented outputs. Two recent studies assessed the ability of GPT-4 with vision technology (GPT-4V) to differentiate between benign nevi and melanomas, but their conclusions were discordant and limited by small datasets without human comparators.^1^^,^^2^ Consequently, a gap remains in our understanding of GPT-4V's capabilities in generating image-based dermatologic diagnoses.

In this diagnostic accuracy study, we configured 3 custom versions of ChatGPT, termed “GPTs,” to analyze dermoscopic images, clinical images, and paired dermoscopic and clinical images, respectively (Supplementary Methods, available via Mendeley at https://data.mendeley.com/datasets/tgf4xjrdgw/2). The STARD reporting and CLEAR Derm consensus guidelines were followed (Supplementary Table I, available via Mendeley at https://data.mendeley.com/datasets/tgf4xjrdgw/2).^3^^,^^4^ Each GPT described discernable dermoscopic and/or gross features, constructed a differential diagnosis, and recommended biopsy or monitoring. Five board-certified dermatologists served as comparators (Supplementary Table II, available via Mendeley at https://data.mendeley.com/datasets/tgf4xjrdgw/2).

A sample of 125 pairs of de-identified images of biopsy-confirmed benign and malignant skin lesions from 118 patients was selected from one dermatologist’s (NG) practice (Supplementary Table III, available via Mendeley at https://data.mendeley.com/datasets/tgf4xjrdgw/2). GPTs were prompted with an image or image pair, accompanied by a one-sentence description of lesion location (Supplementary Fig 1, available via Mendeley at https://data.mendeley.com/datasets/tgf4xjrdgw/2). Dermatologists evaluated 3 sets of PowerPoint slides featuring the same images and descriptions in randomized order, with the addition of distractor photos to mitigate potential exposure bias. Primary outcome measures were sensitivity, specificity, positive predictive value, negative predictive value, and accuracy, calculated based on correct identification of lesions as either benign or malignant. Two-sample proportions z-tests determined the statistical significance of differences between dermatologists and GPT-4V. Secondarily, biopsy recommendations from paired image analyses were assessed, and percentages correct were computed based on both proposed and histologically confirmed diagnoses (Mendeley Supplementary Methods, available via Mendeley at https://data.mendeley.com/datasets/tgf4xjrdgw/2).

GPT-4V demonstrated a sensitivity of 50.8% for dermoscopic-only and clinical-only models, with specificities of 67.8% and 75.8%, respectively. With paired images, sensitivity improved to 66.7%, inferior to that of the dermatologists (87.0%; *P* < .001), and specificity to 79.0%, superior to that of the dermatologists (63.2%; *P* = .024). Similar enhancements in GPT-4V’s positive predictive value, negative predictive value, and accuracy were noted with paired images, reaching 76.4%, 70.0%, and 72.8%, respectively (Table I). Management decisions were similar between GPT-4V and dermatologists, though the model recommended biopsy for histologically confirmed malignant lesions less frequently than any dermatologist (Table II).

**Table I:** Performance of GPT-4V and dermatologists (based on sensitivity, specificity, PPV, NPV, and accuracy) on dermoscopy images only, clinical images only, and both dermoscopy and clinical images

	Dermoscopy			Clinical			Dermoscopy + Clinical		
	GPT-4V	Derm	*P*-value	GPT-4V	Derm	*P*-value	GPT-4V	Derm	*P*-value
Sensitivity	50.8% (32/63)	75.2% (237/315)	**.0017**	50.8% (32/63)	83.5% (263/315)	**<.001**	66.7% (42/63)	87.0% (274/315)	**<.001**
Specificity	67.8% (42/62)	69.7% (216/310)	.88	75.8% (47/62)	57.4% (178/310)	**.01**	79.0% (49/62)	63.2% (196/310)	**.024**
PPV	61.5% (32/52)	71.6% (237/331)	.19	68.1% (32/47)	66.6% (263/395)	.97	76.4% (42/55)	70.6% (274/388)	.47
NPV	57.5% (42/73)	73.5% (216/294)	**.012**	60.3% (47/78)	60.3% (47/78)	1.00	70.0% (49/70)	82.7% (196/237)	**.031**
Accuracy	59.2% (74/125)	72.2% (361/500)	**.0066**	63.2% (79/125)	69.6% (348/500)	.20	72.8% (91/125)	75.6% (378/500)	.595

Bolded values are *P* < .05.

*Derm*, Dermatologists; *GPT-4V*, generative pre-trained transformer-4 with vision technology; *NPV*, negative predictive value; *PPV*, positive predictive value.

**Table II:** Decision to biopsy: GPT-4V versus board-certified dermatologists

	Histologic diagnosis (A/B, %)		Top clinical diagnosis (A/C, %)	
	Benign (*n* = 62)	Malignant (*n* = 63)	Benign	Malignant
Derm 1	18/62 (29.0%)	53/63 (84.1%)	3/42 (7.1%)	68/83 (81.9%)
Derm 2	37/62 (59.7%)	59/63 (93.7%)	10/37 (27.0%)	86/88 (97.7%)
Derm 3	28/62 (45.2%)	57/63 (90.5%)	14/54 (25.9%)	71/71 (100%)
Derm 4	20/62 (32.3%)	56/63 (88.9%)	2/51 (3.9%)	74/74 (100%)
Derm 5	44/62 (71.0%)	60/63 (95.2%)	32/53 (60.4%)	72/72 (100%)
Derm average	**29/62 (46.7%)**	**57/63 (90.5%)**	**12/47 (25.5%)**	**74/78 (94.9%)**
GPT-4V	**20/62 (32.3%)**	**45/63 (71.4%)**	**11/70 (15.7%)**	**54/55 (98.2%)**

The decisions to biopsy were based solely off of results of paired dermoscopy and clinical image assessments. The numerator reflects the number of lesions chosen to biopsy, and the denominator reflects the number of either the histologic diagnosis or the top clinical diagnosis that fall into the benign or malignant category.

Each of the values in the table is representative of the fraction (and associated percentage, in parentheses) of each category of skin lesion (ie, histologically benign, histologically malignant, diagnosed by participant/model as most likely benign, diagnosed by participant/model as most likely malignant) that the dermatologist or model believed should be biopsied. For example, the first bolded value of “29/62 (46.7%)” means that, of 62 histologically-confirmed benign lesions included in this study, the dermatologists (on average) would decide to biopsy 29 of them.

*A*, Lesion recommended for biopsy; *B*, histologic diagnosis; *C*, top clinical diagnosis; *Derm*, dermatologists; *GPT-4V*, generative pretrained transformer.

Limitations of our study include potential variations in image and screen quality, the use of a single-institution sample of 5 dermatologists, and the predominance of Fitzpatrick skin type II in our sample, which may limit generalizability. Regardless, our results highlight GPT-4V's remarkable specificity and information-synthesizing capabilities, evidenced by substantial improvements across all measures with paired images. This suggests the model’s value as a low-cost, accessible clinical supplement. However, GPT-4V falls short of replicating the clinical reasoning and ethical skills that dermatologists cultivate during training.^5^ Our findings underscore the necessity of optimizing clinical information fed into AI diagnostic tools, with an emphasis on augmenting—not supplanting—the nuanced decision-making processes of dermatologists.

## Conflicts of interest

None disclosed.
